# Clinical presentation of body-focused repetitive behaviors in minority ethnic groups

**DOI:** 10.1016/j.comppsych.2021.152272

**Published:** 2021-11

**Authors:** Jon E. Grant, Stephanie Valle, Ibrahim H. Aslan, Samuel R. Chamberlain

**Affiliations:** a Department of Psychiatry and Behavioral Neuroscience, University of Chicago, Chicago, IL, USA; b Department of Psychiatry, Faculty of Medicine, University of Southampton, Southampton, UK; c Southern Health NHS Foundation Trust, Southampton, UK

**Keywords:** Trichotillomania, Skin picking, Race, Ethnicity, Impairment, Comorbidity

## Abstract

**Background:**

Body-focused repetitive behaviors (BFRBs), such as trichotillomania and skin picking disorder, are psychiatric disorders characterized by repetitive grooming that result in hair loss or excoriations. Questions remain as to whether there are racial/ethnic differences in the clinical presentation of BFRBs.

**Methods:**

We recruited 539 adults with DSM-5 trichotillomania or skin picking disorder. Of these, 76 (14.1%) self-identified as Black, Asian, or Minority Ethnic (BAME), while 463 (85.9%) self-identified as white Caucasian (hereafter referred to as non-BAME). BAME and non-BAME participants were compared on demographics, symptom severity, comorbid conditions and psychosocial impairment.

**Results:**

Groups did not differ in terms of age, sex, or education levels. BAME individuals reported significantly more time spent picking or hair pulling per day compared to non-BAME individuals, and were less likely to have received treatment for their BFRB symptoms. Some differences were also found with respect to where on the body people pull and pick from.

**Discussion:**

In general, the clinical profiles of BFRBs appeared similar between those from BAME versus non-BAME backgrounds. However, differences were found in terms of treatments received and an aspect of symptom severity. The findings highlight the need to better understand the heterogeneity of BFRBs including potential health inequalities.

## Highlights

• Few studies have examined associations of race/ethnicity on body focused repetitive behaviors.
• This study found that ethnic minorities experienced greater severity of picking and pulling.
• Ethnic minorities were less likely to have received treatment for trichotillomania or skin picking disorder.

## 1 Introduction

Trichotillomania and skin picking disorder are characterized by excessive grooming leading to hair loss or excoriations, respectively, as well as causing clinically significant impairment or distress [1,2]. These two disorders have been grouped together in a category referred to as body-focused repetitive behaviors (BFRBs). Trichotillomania and skin picking disorder both appear to be fairly common psychiatric disorders with current prevalence rates of 1.7% and 2.1%, respectively [3,4].

Trichotillomania and skin picking disorder are characterized by the repetitive pulling out of one's own hair or picking one's own skin resulting in distress or functional impairment [5]. Most people with trichotillomania commonly pull from the scalp, eyebrows, and eyelashes but any bodily site with hair may be the focus. People with skin picking most frequently pick from their face but as with trichotillomania they may picking from any part of the body. Pulling and picking from multiple sites is common and pulling and picking episodes are usually daily and can last from a few minutes to several hours [5]. Many people report not being fully aware of their pulling or picking behaviors (referred to as “automatic” pulling/picking), whereas “focused” pulling and picking generally occurs when the person intends to correct a hair or skin problem (e.g., remove a hair that is “not right” or picking a perceived bump on the skin as not feeling “smooth enough”) [2,6]. Both behaviors are frequently associated with reduced self-esteem, and increased avoidance of social situations due to shame and embarrassment. Even though trichotillomania and skin picking interfere with a person's quality of life, the majority (about 65% or higher) of individuals never seek treatment [7]. Psychiatric comorbidity is common in BFRBs with anxiety/depressive disorders, obsessive compulsive disorder, post-traumatic stress disorder, and attention deficit hyperactivity disorder being the most common and often the reason people seek treatment [5].

While the majority of research regarding the clinical characteristics of BFRBs has focused on white Caucasian samples, very little research has examined to what extent there are differences in the presentation of BFRBs as a function of racial-ethnic groups. Of the studies examining racial/ethnic differences in BFRBs, there have been only a few in trichotillomania and none published in the area of skin picking disorder. One online survey found that White Caucasian participants (*n* = 1290) were more likely to report trichotillomania symptoms interfered with academic life, whereas Black, Asian, and Minority Ethnic (BAME) individuals (*n* = 103) were more likely to report interference within the domain of home management and were less likely to seek treatment [8]. In a telephone study, black females reported that trichotillomania impairment and severity were positively correlated with symptoms of anxiety [9]. A study of 43 black women found significant correlations between aspects of a participant's racial identity and their affect before, during and after a hair pulling episode [10]. Another study of black students (*n* = 176) compared to non-black students (*n* = 422; which included ethnic minorities and white Caucasians) found that black individuals were more likely to pull hair in response to skin irritation and had higher rates of noticeable hair loss due to pulling [11] (other research failed to find similar results however; see [12]). Finally, one study examined response to behavioral therapy between racial groups (*n* = 15 BAME and 38 non-BAME) and found that although fewer minority participants reported improvement during the internet-based self-help, there were no differences in rates of improvement between groups during the in-person therapy [13].

Although the data on BFRBs and racial-ethnic status are limited and somewhat mixed, racial differences may be particularly relevant to BFRBs. BFRBs often result in noticeable physical or cosmetic problems (e.g., hair loss or thinning, excoriations). Consequently, those with BFRBs often avoid social settings due to appearance-related embarrassment [14]. While skin conditions such as acne, vitiligo, and psoriasis are not psychiatric in nature, they also create noticeable visual abnormalities on those afflicted. Within these conditions, ethnicity has been shown to play a role in self-consciousness (a component of psychosocial impairment) [15]. As such, these studies suggest that ethnicity may play a role in both symptom severity and psychosocial impairment associated with BFRBs.

One approach to more precisely tailor BFRB treatments [13] might be to explore the relationship between ethnicity and clinical presentation of both trichotillomania and skin picking disorder. Are there unique racial differences that should be better understood and ultimately be incorporated into education programs to promote treatment utilization as well as refine and improve treatment approaches? The goal of this study was to better understand how ethnicity may relate to symptom severity and psychosocial impairment in a sample of adults with trichotillomania or skin picking disorder. In light of the current literature including for other disorders, we hypothesized that there would be a difference in symptom severity, co-occurring disorders, and rates of treatment utilization, between BAME and non-BAME individuals.

## 2 Material & methods

### 2.1 Participants

Adults, ages 18–65 with a primary and current DSM-5 diagnosis of trichotillomania or skin picking disorder were enrolled for various research studies over the period from 2009 to 2021. Participants were primarily recruited from a large urban area using referrals and advertisements (online and print). Exclusion criteria included: (1) any neurological or psychiatric conditions that would prohibit completion of questionnaires, and (2) inconsistent or unstable psychotropic medication use or psychotherapy participation within the prior three months.

Data were collected at the University of Chicago following Institutional Review Board approval of the studies and associated consent procedures. Participants were given a comprehensive explanation of the study procedures and were given the opportunity to put forth any questions. After all questions were answered, participants provided written informed consent. This research was carried out in accordance with the principles of the Declaration of Helsinki.

### 2.2 Assessments

Participants were diagnosed utilizing DSM-5 criteria. In the cases where participants were enrolled before DSM-5 criteria, there was sufficient history to be able to transfer to DSM-5 criteria. Baseline demographic information included current age, self-identified race-ethnic grouping, educational background, employment, and marital status. Clinical characteristics were assessed with a semi-structured interview including questions regarding hair-pulling/skin picking behavior.

All participants were screened for co-occurring or lifetime psychiatric disorders using the Mini-International Neuropsychiatric Interview (MINI) [16]. Family medical and psychiatric history were assessed by interview of the proband (no family members were interviewed).

In addition, the following clinical measures were used to assess symptom severity, quality of life, psychosocial dysfunction/impairment, as well as measures related to anxiety, depression, and impulsivity (all measures have previously demonstrated excellent psychometric properties in studies of BFRBs [17]): *Massachusetts General Hospital Hairpulling Scale (MGH-HPS)* is a seven-item Likert-type scale used to assess severity of hair-pulling in several different domains [18]; *Skin Picking Symptom Assessment Scale (SP-SAS)* is a 12-item self-report scale examining severity of picking behavior during the past week [19]; *Clinical Global Impression – Severity scale (CGI-S)* is a 7-item scale used in this study to assess clinical severity of SPD symptoms [20]; *Milwaukee Inventory for Subtypes of Trichotillomania-Adult Version (MIST-A)* assesses intentionality and emotionality [21]; *Milwaukee Inventory for the Dimensions of Skin Picking (MIDAS)* examines automatic and focused picking styles [22]; *Hamilton Anxiety Rating Scale (HAM-A)* [23] provides an overall measure of global anxiety; *Hamilton Depression Rating Scale (HAM—D)* [24] assesses severity of depressive symptoms; *Barratt Impulsiveness Scale 11 (BIS-11)* [25], a self-report questionnaire examines general impulsiveness as well as three second order domains: motor, non-planning, and attentional impulsiveness; and the *Sheehan Disability Scale (SDS)* a self-report measure of functional disability [26].

### 2.3 Data analysis

Participants were classified into two groups: those who self-identified as Black, Asian or Minority Ethnic (BAME) and those who self-identified as white Caucasian (non-BAME). Groups were compared on all measures using *t*-tests or chi-square. This being an exploratory study, and in view of the sample size, statistical significance was defined as *p* < 0.05 uncorrected. All analyses were conducted using JMP Pro software. We decided to pool all BAME individuals due to the small sample sizes in individual ethnic minority groups (Table 1). We have however presented the data on each racial group separately in the Supplement.

**Table 1:** Number of people in individual racial groups in the study. Due to low cell counts for each minority group, the study focused on a pooled analysis (BAME).

Count Total % Col % Row %	Trichotillomania	Skin Picking Disorder	Comorbid TTM & SPD
Caucasian	312 57.88 86.19 67.39	142 26.35 85.03 30.67	9 1.67 90.00 1.94
Mixed Caucasian and Black	1 0.19 0.28 100.00	0 0.00 0.00 0.00	0 0.00 0.00 0.00
Mixed Caucasian and Asian	0 0.00 0.00 0.00	1 0.19 0.60 100.00	0 0.00 0.00 0.00
Black	25 4.64 6.91 73.53	9 1.67 5.39 26.47	0 0.00 0.00 0.00
Latino/Hispanic	6 1.11 1.66 54.55	5 0.93 2.99 45.45	0 0.00 0.00 0.00
Asian	14 2.60 3.87 73.68	4 0.74 2.40 21.05	1 0.19 10.00 5.26
Native American	0 0.00 0.00 0.00	1 0.19 0.60 100.00	0 0.00 0.00 0.00
Other	4 0.74 1.10 44.44	5 0.93 2.99 55.56	0 0.00 0.00 0.00

## 3 Results

The sample consisted of 539 adults with BFRBs, with *n* = 76 (14.1%) being of BAME background. Of the total sample, 362 had trichotillomania (*n* = 50 BAME), 167 had skin picking disorder (*n* = 25 BAME), and 10 had both disorders (*n* = 1 BAME). The demographics of the sample are presented in Table 2. The two groups did not differ in terms of demographic characteristics, except for occupational status.

**Table 2:** Demographic characteristics.

		Non-white or mixed race		White		Statistic	df	*p*-value
		n	M (SD)	n	M (SD)			
Group [%]	TTM	50	[65.8]	312	[67.4]	0.280c	2	0.870
	SPD	25	[32.9]	142	[30.7]			
	Both	1	[1.3]	9	[1.9]			
Age (years)		76	31.5 (10.3)	463	31.6 (9.1)	-0.099 t	537	0.920
Sex [%]	Female	67	[88.2]	413	[89.2]	0.348c	2	0.840
	Male	8	[10.5]	47	[10.2]			
	Intersex	1	[1.3]	3	[0.6]			
Education [%]	Less than high school	2	[4.4]	17	[4.7]	4.366c	5	0.498
	High school grad/GED	7	[15.6]	30	[8.4]			
	Some college	10	[22.2]	73	[20.3]			
	College grad	16	[35.6]	118	[32.9]			
	Graduate school plus	10	[22.2]	118	[32.9]			
Occupation [%]	Full-time student	14	[30.4]	144	[39.7]	20.120c	9	0.017*
	Student and working	3	[6.5]	16	[4.4]			
	Work full-time	10	[21.7]	99	[27.3]			
	Work part-time	7	[15.2]	55	[15.1]			
	Retired	0	[0.0]	10	[2.7]			
	Disability	0	[0.0]	5	[1.4]			
	Unemployed	12	[26.1]	24	[6.6]			

All results are mean (SD) unless otherwise noted.

Statistic: c = Chi-square; t = t-test.

Bold p-value indicates significance at <0.05 with effect size.

In terms of clinical characteristics of the BFRBs, BAME participants reported significantly more minutes in a typical day spent on pulling or picking (137.3 [SD 89.6] minutes each day compared to 84.1 [73.7] minutes; *t* = −3.215; df = 233; *p* = 0.002) in the non-BAME participants. BAME individuals were also significantly more likely to pull or pick at their fingers and feet. Interestingly, paper pencil measures of severity, quality of life, or type of BFRB (automatic, focused) did not differ significantly between groups (Table 3).

**Table 3:** Symptom severity, quality of life, and type of BFRB (automatic, focused).

		Non-white or mixed race		White		Statistic	df	p-value
		n	M (SD)	n	M (SD)			
Pull or pick from multiple sites [%]	No	11	[35.5]	114	[38.3]	0.092c	1	0.761
	Yes	20	[64.5]	184	[61.7]			
Everywhere [%]	No	28	[93.3]	278	[95.9]	0.366c	1	0.545
	Yes	2	[6.7]	12	[4.1]			
Scalp [%]	No	20	[64.5]	158	[53.4]	1.428c	1	0.232
	Yes	11	[35.5]	138	[46.6]			
Eyebrows [%]	No	10	[71.4]	116	[59.8]	0.770c	1	0.380
	Yes	4	[28.6]	78	[40.2]			
Eyelashes [%]	No	10	[71.4]	125	[64.4]	0.289c	1	0.591
	Yes	4	[28.6]	69	[35.6]			
Face Excluding Eyelashes and Eyebrows [%]	No	21	[67.7]	215	[72.6]	0.326c	1	0.568
	Yes	10	[32.3]	81	[27.4]			
Fingers [%]	No	25	[80.7]	274	[92.6]	4.010c	1	0.045*
	Yes	6	[19.3]	22	[7.4]			
Hands [%]	No	28	[90.3]	275	[92.9]	0.254c	1	0.614
	Yes	3	[9.7]	21	[7.1]			
Arms [%]	No	25	[80.7]	251	[84.8]	0.348c	1	0.555
	Yes	6	[19.3]	45	[15.2]			
Torso [%]	No	28	[90.3]	265	[89.5]	0.019c	1	0.889
	Yes	3	[9.7]	31	[10.5]			
Back [%]	No	26	[92.9]	256	[93.1]	0.207c	2	0.902
	Yes	2	[7.1]	18	[6.5]			
Stomach [%]	No	27	[96.4]	269	[97.8]	0.190c	1	0.663
	Yes	1	[3.6]	6	[2.2]			
Pubic [%]	No	28	[90.3]	278	[93.9]	0.536c	1	0.464
	Yes	3	[9.7]	18	[6.1]			
Legs [%]	No	29	[93.6]	252	[85.1]	1.960c	1	0.162
	Yes	2	[6.4]	44	[14.9]			
Feet [%]	No	23	[85.2]	261	[97.8]	7.214c	1	0.007*
	Yes	4	[14.8]	6	[2.2]			
Frequency of pulling or picking (Minutes/day)		23	137.3 (89.6)	212	84.1 (73.7)	−3.215 t	233	0.002*
MGH-HPS Total		58	16.1 (7.2)	345	16.6 (5.3)	0.709 t	401	0.479
MGH-HPS Factor 1		58	8.8 (4.3)	348	8.9 (3.6)	0.205 t	404	0.838
MGH-HPS Factor 2		58	7.3 (3.2)	348	7.4 (2.7)	0.223 t	404	0.824
SP-SAS		18	27.8 (6.6)	113	28.7 (6.4)	0.527 t	129	0.599
CGI Severity Scale		76	4.1 (1.4)	455	4.2 (1.0)	1.411 t	529	0.159
HAM-D		31	5.2 (5.2)	301	4.3 (3.7)	−1.230 t	330	0.219
HAM-A		31	5.6 (5.5)	301	4.3 (3.5)	−1.792 t	330	0.074
SDS		41	9.0 (8.2)	349	10.0.6 (6.8)	1.347 t	388	0.179
QOL T-score		43	43.0 (12.3)	351	43.7 (11.9)	0.372 t	392	0.710
MIDAS Focused		23	17 (6.9)	110	15.8 (6.1)	−0.839 t	131	0.403
MIDAS Automatic		24	17.9 (5.3)	111	16.4 (4.5)	−1.403 t	133	0.163
MIST-A Focused		45	44.3 (17.8)	219	40.4 (14.9)	−1.541 t	262	0.125
MIST-A Automatic		45	25.3 (13.4)	218	23.5 (10.2)	−0.997 t	261	0.320
BIS Attentional Impulsiveness		25	16.1 (4.7)	151	17.2 (4.6)	1.075 t	174	0.284
BIS Motor Impulsiveness		26	20.1 (4.3)	149	21.7 (4.4)	1.755 t	173	0.081
BIS Non-planning Impulsiveness		26	23.5 (6.2)	149	22.0 (6.7)	−1.022 t	173	0.308

All results are mean (SD) unless otherwise noted.

Statistic: c = Chi-square; t = t-test.

Bold p-value indicates significance at <0.05 with effect size.

Abbreviations: MGH-HPS = Massachusetts General Hospital Hairpulling Scale; SP-SAS = Skin Picking Symptom Assessment Scale; CGI = Clinical Global Impression rating scales; HAM-D = Hamilton Depression Rating Scale; HAM-A = Hamilton Anxiety Rating Scale; SDS = Sheehan Disability Scale; QOL = The Quality of Life Questionnaire; MIDAS = Milwaukee Inventory for the Dimensions of Adult Skin Picking; MIST-A = Milwaukee Inventory for Subtypes of Trichotillomania-Adults; BIS = Barratt Impulsiveness Scale.

Non-BAME participants were significantly more likely to have received any treatment for their BFRBs and were significantly more likely to have received medication for their BFRBs compared to BAME participants (Table 4). Rates of current or lifetime co-occurring psychiatric disorders did not significantly differ as a function of group (Table 5).

**Table 4:** Treatment history.

		Non-white or mixed race		White		Chi-square	df	p-value
		n	M (SD)	n	M (SD)			
Past Treatment for TTM/SPD [%]	No	21	[84.0]	142	[55.3]	8.622	1	0.003*
	Yes	4	[16.0]	115	[44.7]			
Previous Meds for TTM/SPD [%]	No	21	[87.5]	178	[69.5]	8.957	3	0.030*
	Yes, helpful	2	[8.3]	64	[25.0]			
	Yes, somewhat helpful	1	[4.2]	1	[0.4]			
	Yes, not helpful	0	[0.0]	13	[5.1]			
Previous Therapy for TTM/SPD [%]	No	24	[96.0]	191	[74.6]	8.630	4	0.071
	Yes, helpful	1	[4.0]	44	[17.2]			
	Yes, somewhat helpful	0	[0.0]	10	[3.9]			
	Yes, not helpful	0	[0.0]	10	[3.9]			
What type of therapy [%]	None	19	[95.0]	169	[80.1]	6.948	30	1.000
	CBT	1	[5.0]	7	[3.3]			
	Habit Reversal Training	0	[0.0]	2	[1.0]			
	Other	0	[0.0]	33	[15.6]			
Other treatments [%]	No	15	[93.8]	154	[97.5]	5.566	5	0.351
	Yes	1	[6.2]	4	[2.5]			

All results are mean (SD) unless otherwise noted.

Bold p-value indicates significance at <0.05 with effect size.

Abbreviations: TTM = Trichotillomania; SPD = Skin-picking disorder.

**Table 5:** Rates of comorbidities.

		Non-white or mixed race		White		Chi-square	df	p-value
		n	M (SD)	n	M (SD)			
Depression [%]	No	48	[64.0]	296	[64.2]	5.087	2	0.079
	Current	14	[18.7]	122	[26.5]			
	Past	13	[17.3]	43	[9.3]			
Anxiety [%]	No	63	[84.0]	380	[82.8]	1.227	2	0.542
	Current	12	[16.0]	75	[16.3]			
	Past	0	[0.0]	4	[0.9]			
OCD [%]	No	73	[97.3]	436	[94.8]	1.051	1	0.305
	Current	2	[2.7]	24	[5.2]			
	Past	0	[0.0]	0	[0.0]			
AUD [%]	No	74	[98.7]	449	[97.6]	0.758	2	0.685
	Current	1	[1.3]	9	[2.0]			
	Pas	0	[0.0]	2	[0.4]			
PTSD [%]	No	74	[98.7]	449	[98.0]	0.375	2	0.829
	Current	1	[1.3]	8	[1.8]			
	Past	0	[0.0]	1	[0.2]			
Psychotic Disorder [%]	No	60	[100.0]	396	[100.0]	0	0	–
	Current	0	[0.0]	0	[0.0]			
	Past	0	[0.0]	0	[0.0]			
Panic Disorder [%]	No	73	[97.4]	447	[97.8]	1.603	2	0.449
	Current	1	[1.3]	9	[2.0]			
	Past	1	[1.3]	1	[0.2]			
Bipolar [%]	No	75	[100.0]	450	[99.4]	0.922	2	0.631
	Current	0	[0.0]	2	[0.4]			
	Past	0	[0.0]	1	[0.2]			
Eating Disorder [%]	No	73	[97.4]	446	[97.2]	2.130	3	0.546
	Current	1	[1.3]	11	[2.4]			
	Past	1	[1.3]	2	[0.4]			
Body Dysmorphic Disorder [%]	No	72	[98.6]	432	[99.1]	0.120	1	0.729
	Current	1	[1.4]	4	[0.9]			
	Past	0	[0.0]	0	[0.0]			
ADD/ADHD [%]	No	28	[93.3]	271	[91.6]	0.120	1	0729
	Current	2	[6.7]	25	[8.4]			
	Past	0	[0.0]	0	[0.0]			
Personality Disorder [%]	No	26	[100.0]	264	[98.5]	0.746	1	0.388
	Borderline PD	0	[0.0]	4	[1.5]			
	Dependent/Narcissistic	0	[0.0]	0	[0.0]			

All results are mean (SD) unless otherwise noted.

Bold p-value indicates significance at <0.05 with effect size.

Abbreviations: AUD = Alcohol use disorder; ADD = Attention deficit disorder; ADHD = Attention deficit hyperactivity disorder.

When controlling for occupational status using least squares regression, time spent picking/pulling remained significantly different between BAME and controls (*p* = 0.005) and there was no significant effect of employment status on time spent picking/pulling (*p* = 0.508).

## 4 Discussion

There have been few studies focusing on associations of race/ethnicity with respect to BFRBs, and this study adds to the literature by demonstrating that there are far more similarities than differences in adults with BFRBs between BAME and non-BAME individuals. However, several differences between the two study groups were found. The striking differences seem to be that at least on one measure, BAME individuals reported experiencing more severe BFRB behavior (as measured by time spent engaging in the repetitive behavior per day). Though there were differences in employment status between the groups, this did not account for the group difference on time spent engaging in the behaviors. Also, groups differed as to the sites of the BFRB behavior. And finally, BAME participants were less likely to receive treatment for their BFRB.

Our finding that minorities were less likely to receive treatment for their BFRB is important. Interestingly, the percentage of BAME participants in the study (14.1%) do not reflect the percentage of ethnic minorities in the urban area from which they were recruited (approximately 55%) and this may reflect a general lack of treatment seeking on the part of minorities with BFRBs, a lack of treatment seeking more specifically from large academic medical centers, and/or poor outreach from clinicians and researchers to minority communities. Neal-Barnett and colleagues [27] have suggested that black individuals with trichotillomania are more likely to seek assistance from their hair care professionals than psychologists and psychiatrists. Mansueto and colleagues [28] further hypothesized that perhaps black individuals with trichotillomania seek treatment less frequently because they might actually be better able to mask the cosmetic consequences of hair pulling. Whether these explanations apply here or equally across racial categories remains unclear. Also, why this lack of treatment seeking would also apply to those with skin picking disorder remains unanswered. The lower levels of having received treatment in people from BAME backgrounds raises issues around access to care and whether there are health inequalities that affect such individuals, which is important for healthcare providers and clinicians to be aware of. There are other potential explanations for these disparities in treatment utilization, such as mistrust and stigma which need further examination. Further research should continue exploring BFRBs in ethnic minorities as it would be valuable to learn more about treatment approaches that may be best suited for BAME individuals. Neal-Barnett and colleagues [8] suggested that treatment for trichotillomania should consider the psychological aspects of being in a minority group. Whether these culture-based interventions should also develop novel cognitive and behavioral strategies remains unknown but could be a useful future target.

There are several limitations to the current study. We focused our analysis on comparing BAME to non-BAME individuals. We acknowledge that this terminology/definition itself has its problems and is not universally accepted; however, we suggest it may be a useful starting point to better understand heterogeneity in the clinical presentations of BFRBs. We did not report or examine variables in different minority groups as the sample sizes would have been too small to be meaningful. Future work may wish to examine whether the clinical presentations of BFRBs differ in specific racial/ethnic groupings, rather than the broader category of BAME. The current study was not sufficiently large to enable such finer-grained analyses. Additionally, the sample coming specifically from study recruitment may have disproportionately impacted the participation of particular subgroups, and since participants are enrolled from various research studies, there may be different demand characteristics, which influence symptom ratings. Although we examined a set of demographic and clinical variables potentially relevant to BFRBs, this study did not comprehensively address all variables that may be relevant to this issue. For example, we did not examine socio-economic status, social norms, inclusivity, or acculturation. The study may have been underpowered to detect group differences with respect to some of the categorical variables that had relatively lower cell counts – this especially applies, for example, to some of the comorbidities. An additional study limitation is that although the study is the first to include skin picking disorder, it is collapsed in with trichotillomania so we still do not have a sense of unique ethnic differences in skin picking disorder. Lastly, the study shows more variables associated with one group versus another, but findings may not apply to other settings – for example, it remains to be seen whether the findings would be similar in non-treatment seeking groups.

In conclusion, this study found that people from BAME backgrounds reported lower levels of receiving treatment, and higher levels of time spent picking/pulling, compared to those from non-BAME backgrounds. The current study cannot identify underlying causal mechanisms because it was cross-sectional; and any relationships are likely to be complex and multifactorial. Future longitudinal studies with larger samples, incorporating socio-demographic, clinical, and cognitive variables, may further our understanding of the complex heterogeneity of BRFBs.

## Funding

This research did not receive any specific grant from funding agencies in the public, commercial, or not-for-profit sectors.

## Disclosures

Dr. Grant has received research grants from the TLC Foundation for Body-Focused Repetitive Behaviors, Otsuka, Biohaven, Promentis, and Avanir Pharmaceuticals. He receives yearly compensation for acting as editor-in-chief of the Journal of Gambling Studies and has received royalties from Oxford University Press, American Psychiatric Publishing, Inc., Norton Press, and McGraw Hill. Ms. Valle report no financial relationships with commercial interests. Dr. Chamberlain receives honoraria from Elsevier for editorial work at Comprehensive Psychiatry; and Neuroscience and Biobehavioral Reviews. Dr. Chamberlain's role in this study was funded by a Wellcome Trust Clinical Fellowship (110049/Z/15/Z). Dr. Chamberlain previously consulted for Promentis. Dr. Aslan and Ms. Valle have no conflicts to disclose.

## Appendix A Supplementary data

Supplementary material Image 1
