# Supplementary material for: Clinical presentation of body-focused repetitive behaviors in minority ethnic groups
Source: Compr Psychiatry. 2021 Nov;111:152272. doi: 10.1016/j.comppsych.2021.152272 (PMC7611816; doi:10.1016/j.comppsych.2021.152272)
Supplement: Supplementary file 1 — Supplementary material [file mmc1.docx]

| **Supplemental Table 1.** Demographic Characteristics By Race | | | | | |  |  |  |  |  |  |  |  |
| --- | --- | --- | --- | --- | --- | --- | --- | --- | --- | --- | --- | --- | --- |
|  | | **Black (n=34)** | | **White (N=463)** | | | **Latino (n=11)** | | **Asian (n=19)** | | **Other (n=12)** | | |
|  |  | **n** | **M (SD)** | **n** | **M (SD)** | | **N** | **M (SD)** | **N** | **M (SD)** | | **N** | **M (SD)** |
| Group [%] | TTM | 25 | [73.5] | 312 | [67.4] | | 6 | [54.5] | 14 | [73.7] | | 5 | [41.7] |
|  | SPD | 9 | [26.5] | 142 | [30.7] | | 5 | [45.5] | 4 | [21.1] | | 7 | [58.3] |
|  | Both | 0 | 0 | 9 | [1.9] | | 0 | 0 | 1 | [5.3] | | 0 | 0 |
| Age (years) | |  | 33.3 (8.4) | 463 | 31.6 (9.1) | | 11 | 29.0 (10.9) | 19 | 29.3 (5.9) | | 12 | 26.8 (10.0) |
| Sex [%] | Female | 32 | [94.1] | 413 | [89.2] | | 9 | [81.8] | 14 | [73.7] | | 12 | [100.0] |
|  | Male | 2 | [5.9] | 47 | [10.2] | | 1 | [9.1] | 5 | [26.3] | | 0 | 0 |
|  | Intersex | 0 | 0 | 3 | [0.6] | | 1 | [9.1] | 0 | 0 | | 0 | 0 |
| Education [%] | Less than high school | 1 | [2.9] | 17 | [4.7] | | 2 | [18.2] | 0 | 0 | | 3 | [25.0] |
|  | High school grad/GED | 21 | [61.8] | 30 | [8.4] | | 2 | [18.2] | 11 | [57.9] | | 0 | 0 |
|  | Some college | 6 | [17.6] | 73 | [20.3] | | 1 | [9.1] | 1 | [5.3] | | 2 | [16.7] |
|  | College grad | 5 | [14.7] | 118 | [32.9] | | 4 | [36.4] | 5 | [26.3] | | 2 | [16.7] |
|  | Graduate school plus | 1 | [2.9] | 118 | [32.9] | | 2 | [18.2] | 2 | [10.5] | | 5 | [41.7] |
| Occupation [%] | Full-time student | 21 | [61.8] | 144 | [39.7] | | 2 | [18.2] | 14 | [73.7] | | 5 | [41.7] |
|  | Student and working | 2 | [5.9] | 16 | [4.4] | | 1 | [9.1] | 0 | 0 | | 0 | 0 |
|  | Work full-time | 4 | [11.8] | 99 | [27.3] | | 1 | [9.1] | 2 | [10.5] | | 3 | [25.0] |
|  | Work part-time | 2 | [5.9] | 55 | [15.1] | | 1 | [9.1] | 2 | [10.5] | | 2 | [16.7] |
|  | Retired | 0 | 0 | 10 | [2.7] | | 0 | 0 | 0 | 0 | | 0 | 0 |
|  | Disability | 0 | 0 | 5 | [1.4] | | 0 | 0 | 0 | 0 | | 0 | 0 |
|  | Unemployed | 5 | [14.7] | 24 | [6.6] | | 6 | [54.5] | 1 | [5.3] | | 2 | [16.7] |
| Age of Onset | |  | 15.0 (9.5) | 282 | 12.8 (7.5) | |  | 9.6 (2.7) |  | 11.0 (6.6) | |  | 10.0 (4.3) |

All results are mean (SD) unless otherwise noted

| **Supplemental Table 2.** Symptom severity, quality of life, and type of BFRB by Race | | | | | | | | | | | |
| --- | --- | --- | --- | --- | --- | --- | --- | --- | --- | --- | --- |
|  | | **Black (N=34)** | | **White (N=463)** | | **Latino (N=11)** | | **Asian (N=19)** | | **Other (N=12)** | |
|  |  | **N** | **M (SD)** | **N** | **M (SD)** | N | **M (SD)** | **N** | **M (SD)** | **N** | **M (SD)** |
| Pull or pick from multiple sites [%] | No | 25 | [73.5] | 114 | [38.3] | 9 | [81.8] | 14 | [73.7] | 8 | [66.7] |
|  | Yes | 9 | [26.5] | 184 | [61.7] | 2 | [18.2] | 5 | [26.3] | 4 | [33.3] |
| Everywhere [%] | No | 34 | [100.0] | 278 | [95.9] | 11 | [100.0] | 19 | [100.0] | 12 | [100.0] |
|  | Yes | 0 | 0 | 12 | [4.1] | 0 | 0 | 0 | 0 | 0 | 0 |
| Scalp [%] | No | 28 | [82.4] | 158 | [53.4] | 9 | [81.8] | 17 | [89.5] | 11 | [91.7] |
|  | Yes | 6 | [17.6] | 138 | [46.6] | 2 | [18.2] | 2 | [10.5] | 1 | [8.3] |
| Eyebrows [%] | No | 31 | [91.2] | 116 | [59.8] | 10 | [90.9] | 19 | [100.0] | 12 | [100.0] |
|  | Yes | 3 | [8.8] | 78 | [40.2] | 1 | [9.1] | 0 | 0 | 0 | 0 |
| Eyelashes [%] | No | 31 | [91.2] | 125 | [64.4] | 11 | [100.0] | 19 | [100.0] | 11 | [91.7] |
|  | Yes | 3 | [8.8] | 69 | [35.6] | 0 | 0 | 0 | 0 | 1 | [8.3] |
| Face Excluding Eyelashes and Eyebrows [%] | No | 29 | [85.3] | 215 | [72.6] | 10 | [90.9] | 17 | [89.5] | 11 | [91.7] |
|  | Yes | 5 | [14.7] | 81 | [27.4] | 1 | [9.1] | 2 | [10.5] | 1 | [8.3] |
| Fingers [%] | No | 31 | [91.2] | 274 | [92.6] | 10 | [90.9] | 18 | [94.7] | **11** | **[91.7]** |
|  | Yes | 3 | [8.8] | 22 | [7.4] | 1 | [9.1] | 1 | [5.3] | 1 | [8.3] |
| Hands [%] | No | 33 | [97.1] | 275 | [92.9] | 10 | [90.9] | 18 | [94.7] | 12 | [100.0] |
|  | Yes | 1 | [2.9] | 21 | [7.1] | 1 | [9.1] | 1 | [5.3] | 0 | 0 |
| Arms [%] | No | 33 | [97.1] | 251 | [84.8] | 10 | [90.9] | 18 | [94.7] | 10 | [83.3] |
|  | Yes | 1 | [2.9] | 45 | [15.2] | 1 | [9.1] | 1 | [5.3] | 2 | [16.7] |
| Torso [%] | No | 32 | [94.1] | 265 | [89.5] | 10 | [90.9] | 19 | [100.0] | 12 | [100.0] |
|  | Yes | 2 | [5.9] | 31 | [10.5] | 1 | [9.1] | 0 | 0 | 0 | 0 |
| Back [%] | No | 33 | [97.1] | 256 | [93.1] | 10 | [90.9] | 19 | [100.0] | 12 | [100.0] |
|  | Yes | 1 | [2.9] | 18 | [6.5] | 1 | [9.1] | 0 | 0 | 0 | 0 |
| Stomach [%] | No | 33 | [97.1] | 269 | [97.8] | 11 | [100.0] | 19 | [100.0] | 12 | [100.0] |
|  | Yes | 1 | [2.9] | 6 | [2.2] | 0 | 0 | 0 | 0 | 0 | 0 |
| Pubic [%] | No | 32 | [94.1] | 278 | [93.9] | 11 | [100.0] | 19 | [100.0] | 12 | [100.0] |
|  | Yes | 2 | [5.9] | 18 | [6.1] | 0 | 0 | 0 | 0 | 0 | 0 |
| Legs [%] | No | 32 | [94.1] | 252 | [85.1] | 11 | [100.0] | 19 | [100.0] | 12 | [100.0] |
|  | Yes | 2 | [5.9] | 44 | [14.9] | 0 | 0 | 0 | 0 | 0 | 0 |
| Feet [%] | No | 32 | [94.1] | 261 | [97.8] | 11 | [100.0] | 18 | [94.7] | **11** | **[91.7]** |
|  | Yes | 2 | [5.9] | 6 | [2.2] | 0 | 0 | 1 | [5.3] | 1 | [8.3] |
| Frequency of pulling or picking (Minutes) | | 34 | 115.4 (75.2) | 212 | 84.1 (73.7) | 11 | 156.0 (120.2) | 19 | 128.6 (78.8) | 12 | 143.3 (83.3) |
| MGH-HPS Total | | 25 | 17.9 (5.4) | 345 | 16.6 (5.3) | 6 | 20.8 (6.3) | 15 | 15.2 (6.6) | 5 | 17.1 (5.3) |
| MGH-HPS Factor 1 | | 25 | 10.1 (3.4) | 348 | 8.9 (3.6) | 6 | 11.4 (4.4) | 15 | 9.6 (2.4) | 5 | 9.3 (2.7) |
| MGH-HPS Factor 2 | | 25 | 8.5 (1.5) | 348 | 7.4 (2.7) | 6 | 9.4 (1.9) | 15 | 8.0 (1.3) | 5 | 7.9 (2.7) |
| SP-SAS | | 9 | 25.1 (5.7) | 113 | 28.7 (6.4) | 5 | 26.6 (8.3) | 5 | 32.3 (4.9) | 7 | 32.7 (6.4) |
| CGI Severity Scale | | 34 | 4.2 (1.0) | 455 | 4.2 (1.0) | 11 | 4.1 (1.8) | 19 | 4.1 (1.4) | 12 | 3.5 (1.9) |
| HAM-D | | 34 | 4.6 (5.0) | 301 | 4.3 (3.7) | 11 | 9.8 (6.8) | 19 | 2.1 (2.8) | 12 | 7.0 (4.2) |
| HAM-A | | 34 | 5.7 (6.9) | 301 | 4.3 (3.5) | 11 | 8.6 (3.3) | 19 | 2.9 (3.3) | 12 | 6.0 (3.9) |
| SDS | | 34 | 10.8 (8.9) | 349 | 10..6 (6.8) | 11 | 7.4 (5.5) | 19 | 6.9 (7.3) | 12 | 8.7 (7.1) |
| QOL T-score | | 34 | 42.3 (15.2) | 351 | 43.7 (11.9) | 11 | 41.1 (11.6) | 19 | 44.8 (8.7) | 12 | 43.8 (12.5) |
| MIDAS Focused | | 25 | 18.0 (7.2) | 110 | 15.8 (6.1) | 6 | 14.8 (8.2) | 15 | 17.3 (8.1) | 5 | 17.4 (6.2) |
| MIDAS Automatic | | 25 | 16.4 (5.2) | 111 | 16.4 (4.5) | 6 | 18.6 (4.3) | 15 | 19.5 (5.0) | 5 | 18.1 (6.7) |
| MIST-A Focused | | 9 | 47.7 (21.5) | 219 | 40.4 (14.9) | 5 | 37.4 (19.9) | 5 | 46.7 (10.0) | 7 | 33.1 (19.6) |
| MIST-A Automatic | | 9 | 29.3 (11.2) | 218 | 23.5 (10.2) | 5 | 19.4 (17.9) | 5 | 24.1 (13.1) | 7 | 22.6 (14.0) |
| BIS Attentional Impulsiveness | | 34 | 13.3 (3.7) | 151 | 17.2 (4.6) | 11 | 17.0 (6.2) | 19 | 19.3 (6.4) | 12 | 17.4 (2.5) |
| BIS Motor Impulsiveness | | 34 | 19.3 (4.1) | 149 | 21.7 (4.4) | 11 | 20.7 (6.5) | 19 | 20.3 (3.2) | 12 | 20.6 (3.6) |
| BIS Non-planning Impulsiveness | | 34 | 22.4 (5.7) | 149 | 22.0 (6.7) | 11 | 26.2 (7.7) | 19 | 20.0 (6.0) | 12 | 24.1 (5.8) |

All results are mean (SD) unless otherwise noted

Abbreviations: MGH-HPS= Massachusetts General Hospital Hairpulling Scale; SP-SAS = Skin Picking Symptom Assessment Scale; CGI= Clinical Global Impression rating scales; HAM-D= Hamilton Depression Rating Scale; HAM-A= Hamilton Anxiety Rating Scale; SDS = Sheehan Disability Scale; QOL= The Quality of Life Questionnaire; MIDAS = Milwaukee Inventory for the Dimensions of Adult Skin Picking; MIST-A= Milwaukee Inventory for Subtypes of Trichotillomania-Adults; BIS= Barratt Impulsiveness Scale

| **Supplemental Table 3.** Treatment history by Race | | | | | | | | | | | |
| --- | --- | --- | --- | --- | --- | --- | --- | --- | --- | --- | --- |
|  | | **Black (n=34)** | | **White (n=463)** | | **Latino (n=11)** | | **Asian (n=19)** | | **Other (n=12)** | |
|  |  | **n** | **%** | **n** | **%** | **n** | **%** | **n** | **%** | **n** | **%** |
| Past Treatment for TTM/SPD [%] | No | 30 | 88.2 | 142 | [55.3] | 11 | [100.0] | 19 | [100.0} | 12 | [100.0] |
|  | Yes | 4 | 11.8 | 115 | [44.7] | 0 | 0 | 0 | 0 | 0 | 0 |
| Previous Meds for TTM/SPD [%] | No | 30 | 88.2 | 178 | [69.5] | 11 | [100.0] | 19 | [100.0] | 12 | [100.0] |
|  | Yes, helpful | 2 | 5.9 | 64 | [25.0] | 0 | 0 | 0 | 0 | 0 | 0 |
|  | Yes, somewhat helpful | 2 | 5.9 | 1 | [0.4] | 0 | 0 | 0 | 0 | 0 | 0 |
|  | Yes, not helpful | 0 | 0 | 13 | [5.1] | 0 | 0 | 0 | 0 | 0 | 0 |
| Previous Therapy for TTM/SPD [%] | No | 33 | 97.1 | 191 | [74.6] | 11 | [100.0] | 19 | [100.0] | 12 | [100.0] |
|  | Yes, helpful | 1 | 2.9 | 44 | [17.2] | 0 | 0 | 0 | 0 | 0 | 0 |
|  | Yes, somewhat helpful | 0 | 0 | 10 | [3.9] | 0 | 0 | 0 | 0 | 0 | 0 |
|  | Yes, not helpful | 0 | 0 | 10 | [3.9] | 0 | 0 | 0 | 0 | 0 | 0 |
| What type of therapy [%] | None | 33 | 97.1 | 169 | [80.1] | 11 | [100.0] | 19 | [100.0] | 12 | [100.0] |
|  | CBT | 1 | 2.9 | 7 | [3.3] | 0 | 0 | 0 | 0 | 0 | 0 |
|  | Habit Reversal Training | 0 | 0 | 2 | [1.0] | 0 | 0 | 0 | 0 | 0 | 0 |
|  | Other | 0 | 0 | 33 | [15.6] | 0 | 0 | 0 | 0 | 0 | 0 |
| Other treatments [%] | No | 33 | 97.1 | 154 | [97.5] | 11 | [100.0 | 19 | [100.0] | 12 | [100.0] |
|  | Yes | 1 | 2.9 | 4 | [2.5] | 0 | 0 | 0 | 0 | 0 | 0 |

Abbreviations: TTM= Trichotillomania; SPD= Skin-picking disorder; CBT=cognitive behavioural therapy

Abbreviations: AUD= Alcohol use disorder; ADD= Attention deficit disorder; ADHD= Attention deficit hyperactivity disorder

| **Supplemental Table 4.**  Rates of comorbidities by race | | | | | | | | | | | |
| --- | --- | --- | --- | --- | --- | --- | --- | --- | --- | --- | --- |
|  | | **Black (n=34)** | | **White (n=463)** | | **Latino (n=11)** | | **Asian (n=19** | | **Other (n=12)** | |
|  |  | **n** | **%** | **n** | **%** | **n** | **%** | **n** | **%** | **n** | **%** |
| Depression [%] | No | 20 | 58.9 | 296 | [64.2] | 7 | 63.6 | 15 | 78.9 | 7 | 58.3 |
|  | Current | 7 | 20.6 | 122 | [26.5] | 3 | 27.3 | 1 | 5.3 | 3 | 25.0 |
|  | Past | 7 | 20.6 | 43 | [9.3] | 1 | 9.1 | 3 | 15.8 | 2 | 16.7 |
| Anxiety [%] | No | 30 | 88.2 | 380 | [82.8] | 8 | 72.7 | 17 | 89.5 | 10 | 83.3 |
|  | Current | 4 | 11.8 | 75 | [16.3] | 3 | 27.3 | 2 | 10.5 | 2 | 16.7 |
|  | Past | 0 | 0 | 4 | [0.9] | 0 | 0 | 0 | 0 | 0 | 0 |
| OCD [%] | No | 33 | 97.1 | 436 | [94.8] | 11 | 100 | 18 | 94.7 | 12 | 100 |
|  | Current | 1 | 2.9 | 24 | [5.2] | 0 | 0 | 1 | 5.3 | 0 | 0 |
|  | Past | 0 | 0 | 0 | [0.0] | 0 | 0 | 0 | 0 | 0 | 0 |
| AUD [%] | No | 34 | 100 | 449 | [97.6] | 11 | 100 | 19 | 100 | 11 | 91.7 |
|  | Current | 0 | 0 | 9 | [2.0] | 0 | 0 | 0 | 0 | 1 | 8.3 |
|  | Past | 0 | 0 | 2 | [0.4] | 0 | 0 | 0 | 0 | 0 | 0 |
| PTSD [%] | No | 33 | 97.1 | 449 | [98.0] | 11 | 100 | 19 | 100 | 12 | 100 |
|  | Current | 1 | 2.9 | 8 | [1.8] | 0 | 0 | 0 | 0 | 0 | 0 |
|  | Past | 0 | 0 | 1 | [0.2] | 0 | 0 | 0 | 0 | 0 | 0 |
| Psychotic Disorder [%] | No | 34 | 100 | 396 | [100.0] | 11 | 100 | 19 | 100 | 12 | 100 |
|  | Current | 0 | 0 | 0 | [0.0] | 0 | 0 | 0 | 0 | 0 | 0 |
|  | Past | 0 | 0 | 0 | [0.0] | 0 | 0 | 0 | 0 | 0 | 0 |
| Panic Disorder [%] | No | 33 | 97.1 | 447 | [97.8] | 11 | 100 | 19 | 100 | 11 | 91.7 |
|  | Current | 0 | 0 | 9 | [2.0] | 0 | 0 | 0 | 0 | 1 | 8.3 |
|  | Past | 1 | 2.9 | 1 | [0.2] | 0 | 0 | 0 | 0 | 0 | 0 |
| Bipolar [%] | No | 34 | 100 | 450 | [99.4] | 11 | 100 | 19 | 100 | 12 | 100 |
|  | Current | 0 | 0 | 2 | [0.4] | 0 | 0 | 0 | 0 | 0 | 0 |
|  | Past | 0 | 0 | 1 | [0.2] | 0 | 0 | 0 | 0 | 0 | 0 |
| Eating Disorder [%] | No | 33 | 97.1 | 446 | [97.2] | 11 | 100 | 19 | 100 | 12 | 100 |
|  | Current | 1 | 2.9 | 11 | [2.4] | 0 | 0 | 0 | 0 | 0 | 0 |
|  | Past | 0 | 0 | 2 | [0.4] | 0 | 0 | 0 | 0 | 0 | 0 |
| Body Dysmorphic Disorder [%] | No | 33 | 97.1 | 432 | [99.1] | 11 | 100 | 19 | 100 | 12 | 100 |
|  | Current | 1 | 2.9 | 4 | [0.9] | 0 | 0 | 0 | 0 | 0 | 0 |
|  | Past | 0 | 0 | 0 | [0.0] | 0 | 0 | 0 | 0 | 0 | 0 |
| ADD/ADHD [%] | No | 34 | 100 | 271 | [91.6] | 10 | 90.9 | 19 | 100 | 11 | 91.7 |
|  | Current | 0 | 0 | 25 | [8.4] | 1 | 9.1 | 0 | 0 | 1 | 8.3 |
|  | Past | 0 | 0 | 0 | [0.0] | 0 | 0 | 0 | 0 | 0 | 0 |
| Personality Disorder [%] | No | 34 | 100 | 264 | [98.5] | 11 | 100 | 19 | 100 | 12 | 100 |
|  | Borderline PD | 0 | 0 | 4 | [1.5] | 0 | 0 | 0 | 0 | 0 | 0 |
|  | Dependent/Narcissistic | 0 | 0 | 0 | [0.0] | 0 | 0 | 0 | 0 | 0 | 0 |
